# Cardiac Angiosarcoma Presenting as Large Pericardial Effusion With Effusive-Constrictive Pericarditis

**DOI:** 10.7759/cureus.60623

**Published:** 2024-05-19

**Authors:** Sonia Vicenty-Rivera, Porfirio E Diaz-Rodriguez, Victor H Molina-Lopez, Jomar N Machuca

**Affiliations:** 1 Cardiology Section, Specialty Medicine Department, Bruce W. Carter Department of Veterans Affairs Medical Center, Miami, USA; 2 Cardiology, Veterans Affairs Medical Center, San Juan, PRI; 3 Internal Medicine, Veterans Affairs Medical Center, San Juan, PRI

**Keywords:** adjuvant chemotheapy, open heart surgery, cardiac tumor, cardiac angiosarcoma, s: malignant pericardial effusion, pericardial effusion

## Abstract

Pericardial angiosarcoma is an extremely rare malignant tumor originating from the endothelial cells of blood vessels within the pericardium. We present a case of a 49-year-old male who presented with symptoms of pericardial effusion and was subsequently diagnosed with pericardial angiosarcoma. This case report highlights the diagnostic challenges and management options associated with this rare entity.

## Introduction

Primary cardiac sarcomas are a type of cancer that grows in connective tissue such as muscle, fat, and vessels and represent 20% of all primary cardiac tumors [1]. Symptoms can be quite non-specific and depend on the tumor's mass effect on the affected cardiac chamber and/or structures. Angiosarcomas develop from abnormal cells in the lining of blood or lymphatic vessels. This type of tumor is extremely rare. We can further divide them into primary cardiac angiosarcoma (PCA), which originate in the heart, and secondary cardiac angiosarcomas, which metastasize to the heart. Primary cardiac angiosarcomas are the most common malignant primary cardiac tumor, even though rare. Ninety percent of these tumors develop in cells that line the right atrium, invading the inferior vena cava and the tricuspid valve. On rare occasions (<5%), angiosarcomas can affect the left cardiac chambers and even coronary arteries [2]. This type of tumor is typically very aggressive in nature when compared to other sarcomas of the heart such as spindle cell sarcoma, undifferentiated pleomorphic sarcoma, and intima sarcomas. They usually grow within the myocardial wall, but they can project into or fill the atrial chamber and even invade the vena cava and tricuspid valve. According to Butany and Yu, the pericardium is involved in about 61% of right-sided cardiac angiosarcomas with cardiac tamponade and pericardial effusions seen as the most common clinical presentation [3]. The prognosis is poor, and most patients die within months of diagnosis, but in cases where metastasis has not yet developed, surgical treatment is usually preferred when feasible. In lieu of angiosarcoma's rarity, standardization for early diagnosis is difficult and not well defined. In this article, we highlight the case of a patient with primary cardiac angiosarcoma who presented with recurrent large pericardial effusion.

## Case presentation

**Figure 1:**
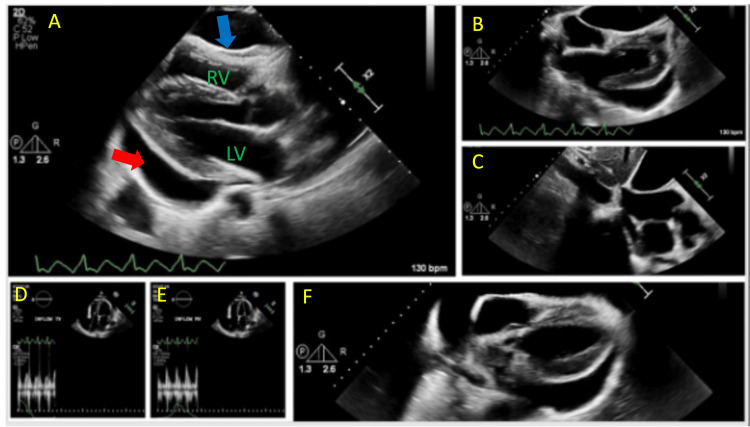
TTE showing a large circumferential pericardial effusion with right ventricle invagination (A, B, F) and plethoric inferior venal cava. Clear pericardial content. No significant mitral (D) nor tricuspid inflow (E) variation with respiration. RV: Right ventricle LV: Left Ventricle TTE: Transthoraric echocardiogram

A 49-year-old man with a history of hypertension, recurrent left-sided myxoid liposarcoma, and prior pericardial effusion with tamponade (treated with pericardiocentesis two months earlier) presented for follow-up transthoracic echocardiogram. Large pericardial effusion had recurred; it was suspected to be secondary to viral pericarditis (Figure 1).

**Figure 2:**
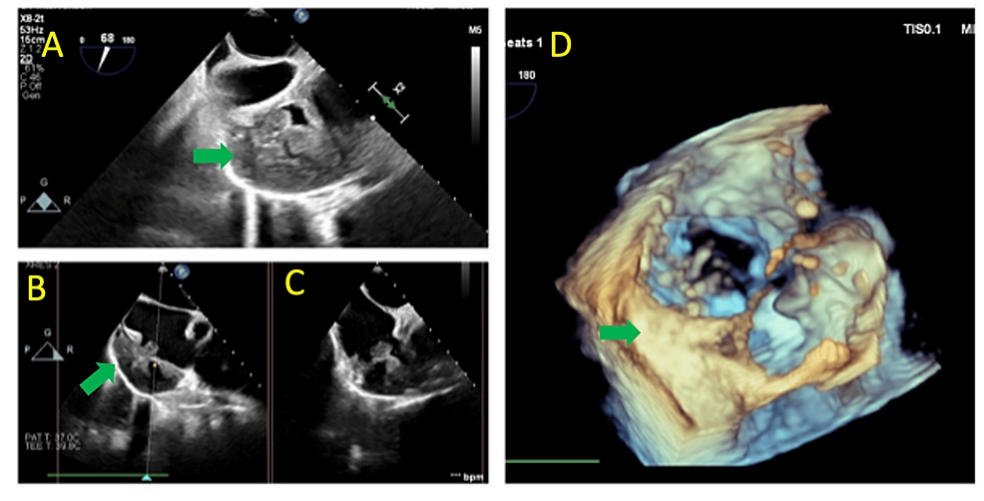
2D TEE (A,B,C) and 3D TEE imaging (D) showing an isoechoic lobulated structure (Green arrow) in the right atrium just above the tricuspid valve suggestive of a mass/thrombus. It measures 4.8cm x3.7cm. In the mass/thrombi there is a hypoechoic avascular structure indicative of cyst or fluid collection measuring 2.3cm x1.1cm. A loculated moderate pericardial effusion is noted measuring 1-2 cm. TEE: Transesophageal echocardiogram Green Arrow: right atrium mass

He reported dyspnea on exertion and pleuritic chest pain, leading to an emergency room visit. EKG showed atrial flutter with a rapid ventricular response. He was admitted for further evaluation. Chest CT was performed which suggested an atrial perforation and an interdisciplinary discussion between cardiothoracic surgery, radiology and cardiology was held. He underwent a limited anterior thoracotomy, pericardiotomy, and partial pericardiectomy. Approximately 1300 ml of bloody fluid was drained from the pericardial space. During surgery, transesophageal echocardiography revealed a mass in the right atrium (Figure 2).

**Figure 3:**
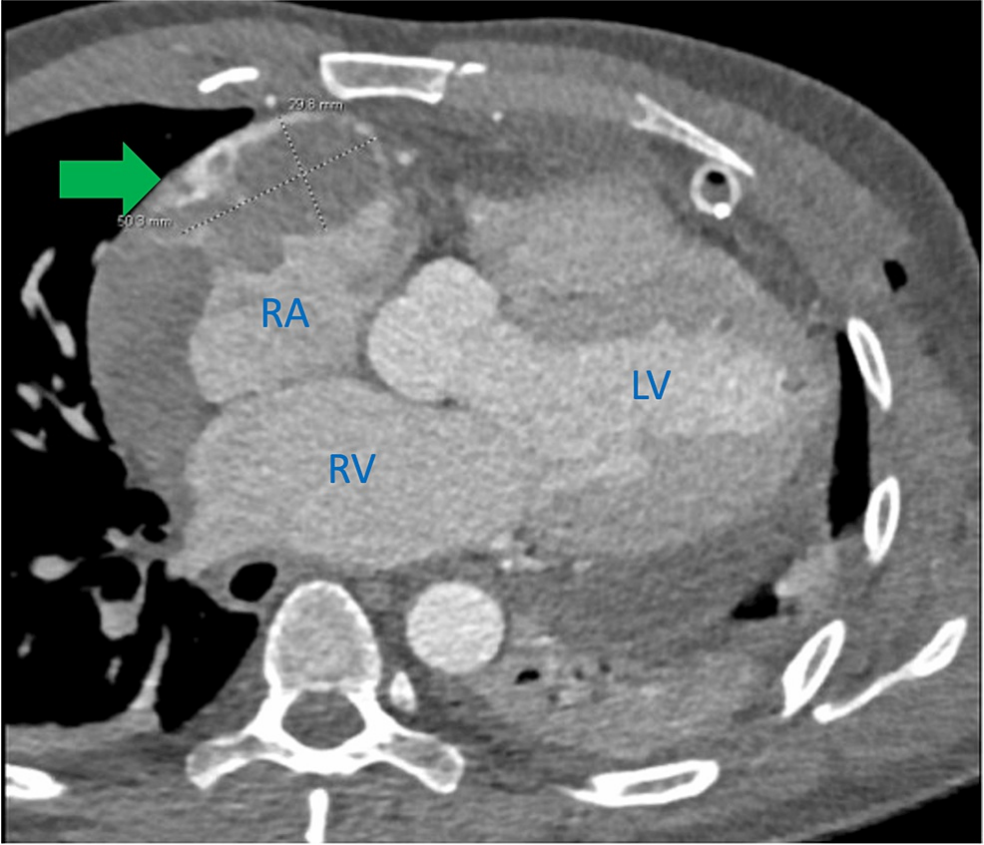
CTA post pericardial window showing an abnormal irregular contour of the right atrial free wall. An adjacent not enhancing hypodense mass (green arrow) measuring 5.0 cm x 2.9 cm surrounded by contrast is also evidenced which is consistent with right atrial rupture contained by a hematoma. CTA: computed tomography angiography Green arrow: hypodense mass in right atrium free wall RA: right atrium RV: right ventricle LV: left ventricle

**Figure 4:**
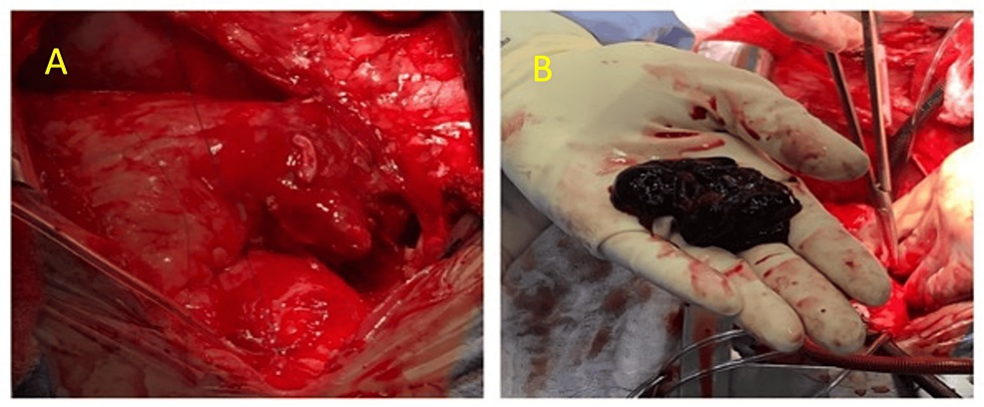
Right atrial hemorrhagic and necrotic tumor fragments with irregular border measuring 4.2x3.2x0.6 cm of dark red or brown color.

A gated chest CT scan was then performed, suggesting a possible contained right atrial rupture with a thrombus (Figure 3). Prompt evaluation and cardiothoracic surgery confirmed the presence of an atrial mass. The mass was partially excised for biopsy and sent for histopathological analysis. Complete surgical removal was not feasible due to extensive involvement of the pericardium. Therefore, debulking surgery was performed (Figure 4).

**Figure 5:**
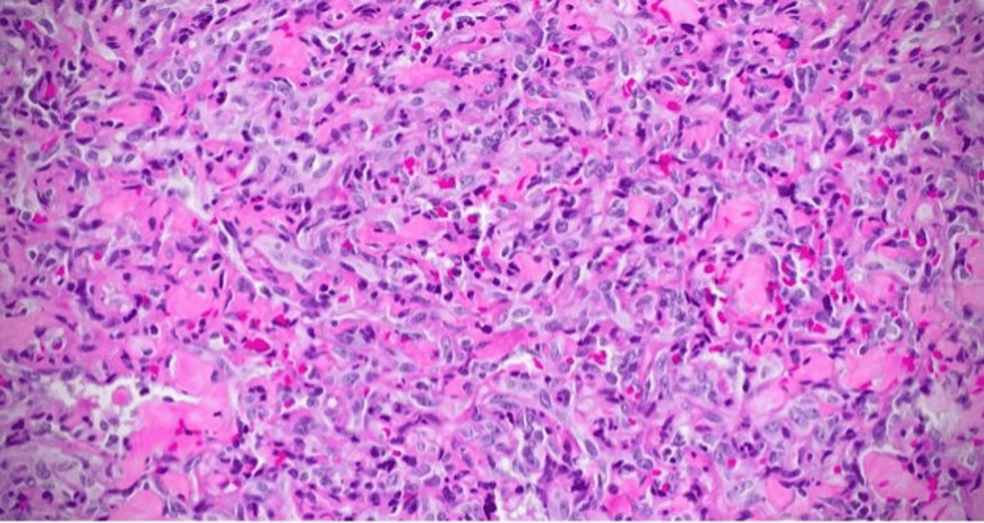
Pathology showing a moderately differentiated angiosarcoma.

**Figure 6:**
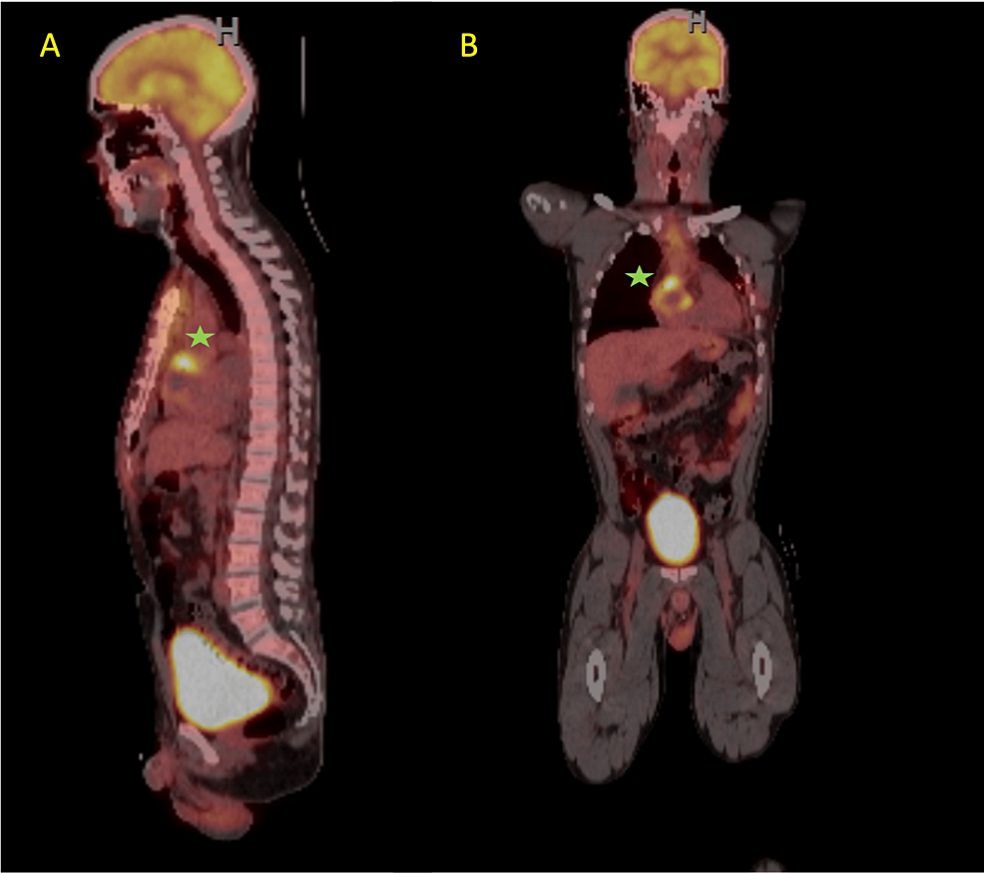
PET 1630 FDG with a focus of severe increased FDG concentration (Star*) with a relatively large central focal defect localized on the free wall of the right atrial area of the myocardium. FDG: fluorodeoxyglucose PET: positron emission tomography

Biopsy results showed moderately differentiated angiosarcoma, with positive immunohistochemical staining for FLI-1, muscle-specific actin (MSA), smooth muscle actin (SMA), PHH3, CD34, and CD31 (Figure 5). A positron emission tomography (PET) CT scan was sent to determine distant metastasis and did not show evidence of metastasis involving any other organ (Figure 6).

**Figure 7:**
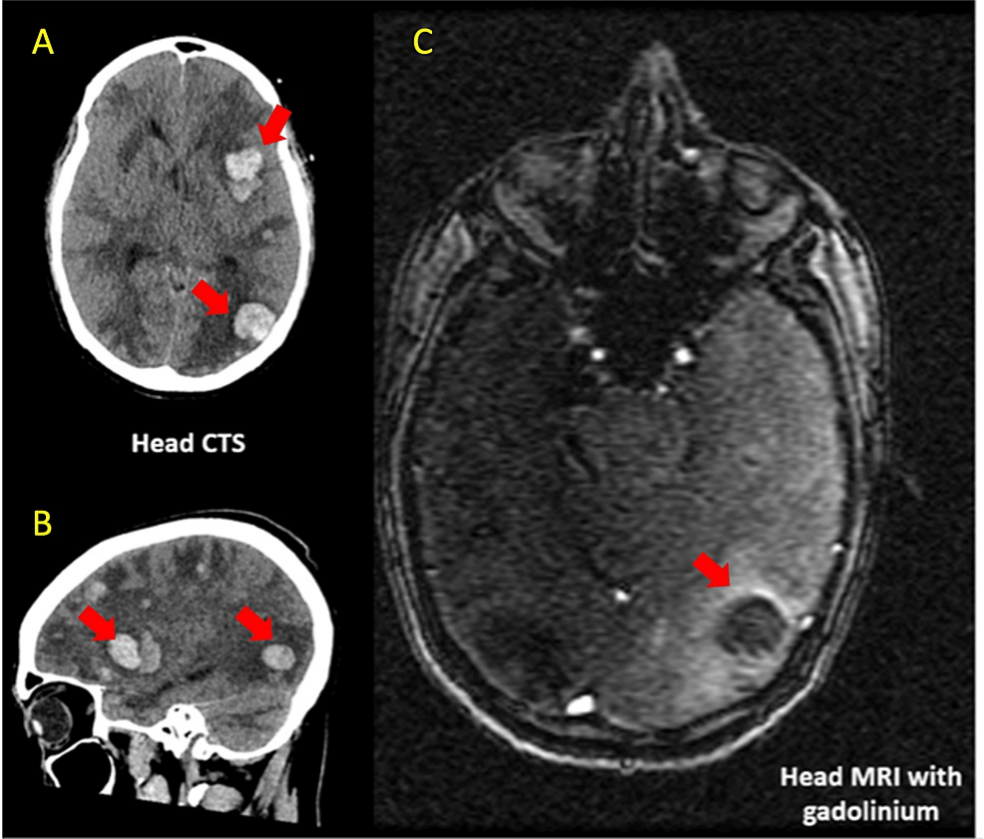
Head CT and MRI with metastatic lesions scattered throughout the supratentorial brain with associated generalized vasogenic edema with effacement of the supratentorial and infratentorial sulci. CT: computer tomography MRI: magnetic resonance imaging Red arrows: metastatic lesions

Therefore, there was consideration for complete cardiac resection and orthotropic heart transplant (OHT). Nevertheless, a second cardiothoracic surgery was requested to consider re-evaluation or OHT. After evaluation it was understood the patient was a poor candidate for open heart surgery as the mass was not amenable for complete resection and there were no clean resection borders (R3). These made him a poor candidate for OHT due to high risk for later metastasis. Hence, it was decided the patient should treated with AIM 60/6 (Adriamycin, ifosfamide and mesna) as adjuvant therapy after debulking heart surgery. The patient received five of the six cycles of AIM chemotherapy when he was hospitalized secondary to anemia. During hospitalization a head CT demostrated numerous hemorrhagic brain metastases with associated vasogenic edema not amenable for surgical intervention. Hence, the patient was treated supportively with medications to reduce vasogenic edema with later neurological status deterioration and demised secondary to herniation and displacement of the thalami, midbrain and tonsillar (Figure 7). 

## Discussion

Primary cardiac tumors are rare with frequency of 0.001% to 0.03%. About 25% of these tumors have a malignant component [1]. Patients with primary cardiac tumors usually remain clinically silent until they reach an advanced stage. Its symptoms can vary depending on the area the tumor affects. Thus, they are often found accidentally by echocardiography when they cause clinical symptoms like those described in our case report. Most malignant heart tumors are sarcomas, but they are still extremely rare. Primary cardiac sarcomas are tumors that generally do not produce symptoms until they are locally advanced and invading adjacent cardiac structures. These delayed manifestations are nonspecific and include dyspnea, chest pain, cardiac arrhythmias, congestive heart failure secondary to obstruction of blood flow as well as systemic symptoms. 

There are limited studies describing the prevalence of PCA. In those, the prevalence of PCA appears to be more likely to present in patients less than 65 years old. Putnam et al. found in a retrospective series that the median age of cardiac sarcoma patients was diagnosed in the fourth and fifth decades of life [4-9]. Furthermore, in terms of gender predisposition, Hamini et al. found a slight male predominance. Nevertheless, most previous studies have found that malignant cardiac tumors are equally prevalent in both genders [2].

Primary cardiac angiosarcomas pose significant diagnostic challenges mostly because of its late presentation and variable symptoms. Its presentation can mimic other pericardial disorders, leading to delays in medical diagnosis. In this case, the patient presented with symptoms of dyspnea on exertion and pleuritic chest pain secondary to recurrent large pericardial effusion with effusive-constrictive pericarditis and difficult-to-control atrial fibrillation. In a retrospective study, Chen et al. analyzed 12 cases of pathologically confirmed cardiac angiosarcoma [10]. In this study it was noticed that none of the angiosarcomas were visible on transthoracic echocardiography, requiring multi-imaging cardiac evaluation for further delineation and description of tumor features which was the situation in our patient. In their analysis, it was noticed that primary cardiac angiosarcoma often involves the right side of the heart with infiltration of peripheral structures. CT features include typical inhomogeneous density on unenhanced scans and heterogeneous centripetal enhancement on enhanced scans. A cauliflower-like appearance on both T1- and T2-weighted MRI is common. Also, other authors have described two most types of angiosarcomas such as a well-defined mass protruding into a cardiac chamber and the right atrium and a diffusely infiltrative mass extending along the pericardium type [11]. Our patient presented with a combination of both findings with cardiac CT describing a low attenuation right atrial mass but also obliteration of the pericardial space with hemorrhagic and necrotic debris. Thus, multi-imaging cardiac modalities, like cardiac CT and 2D, 3D-echocardiography as in our case and cardiac magnetic resonance (CMR) are crucial in establishing the diagnosis. 

In terms of cardiac arrhythmias in patients with primary cardiac tumors, the malignancies are rarely the main etiology for arrhythmias. In the presence of a cardiac tumor, they can cause a spectrum of arrhythmias which can vary from low-grade ectopic beats to incessant atrial and ventricular tachyarrhythmias and even sudden cardiac death. Our patient arrived with dyspnea symptoms along with incessant and difficult-to-control atrial fibrillation requiring high oral dose beta-blockers and IV amiodarone for rate control [12]. Tumor invasion results in abnormalities in the conduction system tissue, therefore, surgical resection might control rhythm abnormalities. In our case this could have a combination of debulking surgery and pericardial fluid drainage. 

Surgical resection remains the treatment of choice for pericardial angiosarcoma, whenever feasible which will also provide accurate tissue diagnosis [13]. Tissue diagnosis is paramount with multiple vascular markers most common of which include BNH9 (monoclonal antibody; positive in 72%), CD31 (transmembrane glycoprotein; positive in 90%), CD34 (hematopoietic progenitor cell antigen; positive in 50-74%), cytokeratin (intermediate filament; positive in 35%), FLU-1 (ETS family transcription factors; positive in 100%), Ki67 (proliferation index; high in 83%), p53 (tumor suppressor; positive 20%), vimentin (intermediate filament; positive), and von Willebrand factor (positive). In our case, the resected tissue was consistent for moderately differentiated angiosarcoma with positive immunohistochemical stains for FL1, MSA, SMA, PHH3, CD34, and CD31. In cases of localized primary cardiac sarcomas, complete resection with microscopic margin (R0) is required to improve its prognosis. However, angiosarcomas have an aggressive nature with rapid disease progression with most patients found frequently with positive surgical margins making complete resection challenging due to the infiltrative nature of the tumor with microscopically positive margins (R1) or partial resection not having any benefit from surgery. Therefore, to decrease risk of local recurrence, adjuvant chemotherapy is recommended to target any residual disease and prevent distant metastasis [14].

The role of heart transplantation remains controversial and was considered as the last surgical resort in the hope of R0 resection of tumors that are deemed unresectable with conventional surgical techniques. Li et al. presented a compilation of 46 patients all diagnosed with sarcomas, six of which were from his intuition and 40 patients from literature. He compared the results to the results of seven patients from his institution who did not undergo heart transplant. Among the transplanted group, the most common subtype was angiosarcoma seen in 30% of them (N=14). In the group receiving heart transplantation for primary cardiac sarcomas the overall survival was as follows: 61% +/- 7% at one year; 44% +/- 8% at two years; 26% +/- 8% at five years after heart transplant with a median survival of 16 months (2-112 months). In this study group, survival relied directly on tumor grade [15]. The fast local infiltration and early metastases contribute to the poor results of current therapies. Furthermore, survival failed to improve despite neoadjuvant or adjuvant chemotherapy after a heart transplant. 

## Conclusions

Pericardial angiosarcoma is an exceptionally rare malignancy that can present with various clinical manifestations. It often mimics other pericardial disorders, leading to delays in diagnosis. Imaging modalities, such as CT and echocardiography, are crucial for establishing the diagnosis.
